# Transplanting a Microbial Organ: the Good, the Bad, and the Unknown

**DOI:** 10.1128/mBio.00572-16

**Published:** 2016-05-03

**Authors:** Dionysios A. Antonopoulos, Eugene B. Chang

**Affiliations:** a Department of Medicine, University of Chicago, Chicago, Illinois, USA; b Biosciences Division, Argonne National Laboratory, Argonne, Illinois, USA

## Abstract

Fecal microbiota transplantation (FMT) has received increased attention as a therapy for correcting intestinal dysbiosis and restoring a state of health in patients suffering from either recalcitrant infection by *Clostridium difficile* or more complex disease states, such as inflammatory bowel disease (IBD). The “gut microbial organ” from the donor that is used in these transplants may serve to transfer genetic material between donor and recipient via virus-like particles, specifically bacteriophages, that infect the bacterial component of the microbiota. The recently published study by Chehoud et al. provides evidence for not only the transfer of bacteriophages during FMT but also the transfer of multiple populations of bacteriophages to recipients from the donor microbiota used (C. Chehoud et al., mBio 7:e00322-16, 2016, http://dx.doi.org/10.1128/mBio.00322-16). While the clinical significance of these findings remains unclear, nothing short of a diligent and persistent effort is needed to define the intended and unintended consequences of FMT.

## COMMENTARY

Fecal microbiota transplantation (FMT), which was first alluded to in the 4th century in China, is the transfer of a donor’s gut microbiota to a recipient with the intent of correcting intestinal dysbiosis and restoring states of health (1, 2). Its feasibility, effectiveness, and putative safety in treating medically refractory cases of antibiotic-associated colitis caused by *Clostridium difficile* have been shown, capturing the attention and imagination of the press, public, and medical profession (3–7). Not surprisingly, a wider application of FMT is now being discussed for other disorders where intestinal dysbiosis has been implicated, including complex immune diseases (inflammatory bowel diseases, type I diabetes, allergic diseases), neurologic diseases (autistic spectrum disorders, Parkinsonism), and metabolic diseases (type II diabetes, obesity) (8). Adding to the hype, commercial sources of FMT preparations as well as do-it-yourself instructions on the internet are becoming increasingly available (9) and, in some cases, have led to unsupervised promiscuous use. In this era of precision medicine, we have the opportunity to develop microbiome-based interventions that can restore health-promoting states of host-microbe interactions. Unfortunately, considering the tremendous interpersonal differences in gut microbial community structure and function between individuals, we have not been able to clearly define a “healthy” gut microbiome. There is almost certainly a multifactorial basis for these differences that are dependent on host assembly rules and conditions, environment and dietary factors, and inherent mechanisms of community selection among microbes. Thus, clinical studies, both randomized control and prospective, are very much needed to provide clarity on which situations FMT has merit, efficacy, and safety and on what factors contribute to a negative as well as a positive clinical outcome.

The study by Chehoud et al. is a step in that direction, by providing evidence that one major component of FMT, bacteriophages, is readily transferred to recipients from the donor microbiota (10). Furthermore, those authors detected elements associated with multiple donor viruses in the same recipient sample. The transfer of multiple viral lineages between individual hosts indicates that these FMT “hitchhikers” may provide an additional route for the transfer of genetic (and functional) diversity between hosts and their “gut microbial organs.” Like any other organ in the body, perturbations and dysfunction of the microbial organ can have widespread consequences and cause or contribute to disease. In the special situation of *C. difficile* colitis, where a patient’s microbial organ is essentially absent, replacement with a donor microbial organ has a strong rationale and proven efficacy. However, in most other cases, the donor microbiota would have to restore or supersede functional problems of the existing microbiota, a task that is both formidable and unpredictable. Like “traditional organ” transplants, potential problems with pathogens, the functionality of donor microbiota, and “rejection” arising from microbe-microbe and microbe-host incompatibilities need to be taken into consideration. The work presented by Chehoud et al. highlights a unique feature of the “gut microbial organ” that should be monitored as well, and it provides a framework for how to do this (10).

This study looked at a single healthy adult donor and three pediatric ulcerative colitis recipient patients who received 22 to 30 FMT treatments over the course of 6 to 12 weeks. Samples collected from the donor and recipients (before, during, and after FMT treatment) were enriched for virus-like particles (VLPs) and analyzed using metagenomic-enabled approaches. This strategy for enriching VLPs from samples up front allowed for a more detailed analysis, since the recovered sequences and functional gene “payload” could be directly ascribed to the enriched fraction of metagenomic data. The initial comparisons of VLP-derived sequences with reference databases yielded a low fraction of aligned reads (average of 17.9%), highlighting their generally underdescribed nature. The investigators also assembled the VLP-enriched fraction of sequence data to provide genomic context (synteny) for functional genes that were then annotated using several protein databases (including ACLAME, CDD, and others [10–12]). Despite these efforts, they were only able to annotate an average of 7.3% of the detected proteins (based on predicted open reading frames), which also highlights the difficulties associated with ascribing functions to VLPs. Prominent conserved protein domains found were phage-associated structural components (e.g., capsids, tails), but domains associated with effector proteins were also detected, including bacteriocins, beta-lactamases, and reverse transcriptases. Despite the low success for annotating functional proteins, the investigators were able to produce circular contigs by assembling the reads, indicating sequencing of full viral genomes. As a result, many contigs could be ascribed to probable viral families (the majority being tailed phages, including those of the *Myoviridae*, *Podoviridae*, and *Siphoviridae* families). Furthermore, by constructing longer contigs (>3 kb), they were able to track the transfer of phage between donor and recipient during FMT. They additionally supported these proposed transfers by using quantitative PCR targeting four specific contigs detected in at least two of the three recipient patients. More interesting was the detection of conserved protein domain families that correlated with the frequency of transmission. These included initiator replication proteins and lambda integrases, which are indicative of a temperate replication strategy. Likewise, based on the assembled contigs, the viral family that was preferentially transferred between donor and recipient was the *Siphoviridae* (which includes the phages λ, 434, and P22), known to be rich in temperate phages. The fact that numerous temperate phages were transferred suggests a common strategy used by phages to optimize their transmission and dispersal between hosts. By observing and tracking the transfer of bacteriophage populations between individuals, the study authors proposed that the transfer of bacteriophage populations is a general characteristic of FMT. However, the longer-term clinical significance of these findings remains uncertain. Though the recipient patients were monitored throughout the course of the study and were found to be symptom-free for at least 4 weeks, all three eventually relapsed and reverted to therapy with immunomodulators.

While many have touted FMT as generally safe as a therapeutic approach, this notion is based on rather limited clinical observations—mostly in subjects with *C. difficile* colitis. The efficacy of FMT in patients with ulcerative colitis is less clear, in part because of the large differences in method, frequency, and source of microbiota administered, compounded by the imprecision of measures to assess outcome, durability, and safety. Moreover, as each person’s gut microbial organ is highly individual, blinded matches of donor FMT to recipient are likely to be met with limited success in treating many complex immune and metabolic diseases, where host factors cannot be easily changed and environmental influences are often obscure and persistent. The malleability of the gut microbial organ provides an opportunity for personalized therapy, but more information is needed to effect this change reproducibly and predictably. This has been challenging with respect to characterizing and studying the “gut microbial organ,” as we are currently at the limit of technology and knowledge, allowing us to look only where the light is (the “streetlight effect”). As highlighted in their manuscript, examples included incomplete annotation resources and, worse yet, incomplete functional characterization. DNA sequencing-enabled approaches to studying microbial communities and their array of populations have been augmented by bioinformatics tools for annotating and tracking specific sequences (and, by proxy, organisms and/or their gene sets). However, there is a current lag in the study of viral/phage elements, owing to a much less structured set of reference data and databases. Recognizing that bacteriophages are a characteristic of FMT and have the potential to alter the composition, features, and functions of the microbiota underscores how ill-defined the “gut microbial organ” currently is. The report by Chehoud et al. serves as a reminder that systematic efforts are sorely needed for understanding baseline bacteriophage dynamics in the gut in order to identify and circumvent problems associated with use of the gut microbiota itself as a therapy for treating disease.
